# Perinatal mesenchymal stromal cells of the human decidua restore continence in rats with stress urinary incontinence induced by simulated birth trauma and regulate senescence of fibroblasts from women with stress urinary incontinence

**DOI:** 10.3389/fcell.2022.1033080

**Published:** 2023-01-18

**Authors:** Paz De La Torre, María Jesús Pérez-Lorenzo, Álvaro Alcázar-Garrido, Jennifer Collado, Mario Martínez-López, Laura Forcén, Ana R. Masero-Casasola, Alicia García, Mª Carmen Gutiérrez-Vélez, José Medina-Polo, Eloy Muñoz, Ana I. Flores

**Affiliations:** ^1^ Regenerative Medicine Group, Research Institute Hospital 12 de Octubre (imas12), Madrid, Spain; ^2^ Pathology Department, Hospital Universitario 12 de Octubre, Madrid, Spain; ^3^ Obstetrics and Gynecology Department, Hospital Universitario 12 de Octubre, Madrid, Spain; ^4^ Male’s Integral Health Group, Urology Department, Research Institute Hospital 12 de Octubre (imas12), Hospital Universitario 12 de Octubre, Madrid, Spain

**Keywords:** stress urinary incontinence, vaginal distention, perinatal mesenchymal stromal cells, myofibroblast, senescence, extracellular matrix remodeling, regeneration, secretome

## Abstract

Stress urinary incontinence (SUI) is a condition that causes the involuntary loss of urine when making small efforts, which seriously affects daily life of people who suffer from it. Women are more affected by this form of incontinence than men, since parity is the main risk factor. Weakening of the pelvic floor tissues is the cause of SUI, although a complete understanding of the cellular and molecular mechanisms of the pathology is still lacking. Reconstructive surgery to strengthen tissue in SUI patients is often associated with complications and/or is ineffective. Mesenchymal stromal cells from the maternal side of the placenta, i.e. the decidua, are proposed here as a therapeutic alternative based on the regenerative potential of mesenchymal cells. The animal model of SUI due to vaginal distention simulating labor has been used, and decidual mesenchymal stromal cell (DMSC) transplantation was effective in preventing a drop in pressure at the leak point in treated animals. Histological analysis of the urethras from DMSC-treated animals after VD showed recovery of the muscle fiber integrity, low or no extracellular matrix (ECM) infiltration and larger elastic fibers near the external urethral sphincter, compared to control animals. Cells isolated from the suburethral connective tissue of SUI patients were characterized as myofibroblasts, based on the expression of several specific genes and proteins, and were shown to achieve premature replicative senescence. Co-culture of SUI myofibroblasts with DMSC *via* transwell revealed a paracrine interaction between the cells through signals that mediated DMSC migration, SUI myofibroblast proliferation, and modulation of the proinflammatory and ECM-degrading milieu that is characteristic of senescence. In conclusion, DMSC could be an alternative therapeutic option for SUI by counteracting the effects of senescence in damaged pelvic tissue.

## 1 Introduction

Stress urinary incontinence (SUI) is a pathological condition characterized by the involuntary loss of urine due to simple efforts such as sneezing, laughing or coughing (Norton and Brubaker, 2006). SUI is the most common form of incontinence worldwide and affects more women than men, with an estimated 35-fold higher prevalence in women of the same age groups (Maral et al., 2001). SUI is not only a medical problem but also a socio-economic problem, given the high percentage of the female population affected and the serious deterioration in their quality of life with global repercussions on personal and work life. The prevalence of SUI in the US is estimated to be 26% (Lee et al., 2021) while in European countries it ranges from 23% in Spain to 42% in the UK (Hunskaar et al., 2004). Annual SUI costs, including direct and indirect costs, are estimated to be close to €10 billion in Europe (Milsom et al., 2014), although the costs of diagnosis, treatment, and follow-up of patients with SUI vary depending on the countries (Zwolsman et al., 2019). The main risk factors for the appearance of SUI are pregnancy and vaginal delivery, although aging, decreased estrogen in menopause and obesity also predispose to SUI. Urinary continence is possible by virtue of a complex mechanism by which urethral resistance counteracts intravesical pressure. In SUI, urethral resistance is insufficient to remain closed and prevent urine leakage when intra-abdominal pressure increases, and this is thought to be primarily due to direct damage to the urethral sphincter and/or weakening of the supporting structures causing hypermobility of the urethra (Gill et al., 2010).

The connective tissue that houses the posterior wall of the urethra and the anterior wall of the vagina is part of the endopelvic fascia and consists of a dense extracellular (ECM) regulated by the resident fibroblasts (Herschorn, 2004). This connective tissue is described as functioning like a sling or a hammock where the urethra would be compressed as intra-abdominal pressure increases. A deficiency in this structure would prevent the complete closure of the urethra and could contribute to SUI (Ashton-Miller and DeLancey, 2007), and several studies have shown alterations in the metabolism of collagen and elastin in periurethral connective tissues of patients affected by SUI (Chen et al., 2006; Chen and Yeh, 2011). Aging, as well as the hormonal decay of menopause are known causes that lead to changes in the composition and structure of the ECM in the anterior wall of the vagina, resulting in mechanical alterations (Falconer et al., 1996; Lin et al., 2007) strongly related to pelvic floor disorders (Gardella et al., 2022). In pregnancy there is an intensive remodeling of the pelvic support structures to allow childbirth, and degenerative processes have been described that affect these structures as a consequence of mechanical stretching and ischemia in vaginal delivery (Ashton-Miller and Delancey, 2009). Placement of a sling made of synthetic or autologous materials to reinforce weakened native tissues is considered the gold standard for SUI surgery. However, this intervention is frequently associated with post-operative complications such as pain, voiding dysfunction, vaginal or bladder perforation, and failure in long-term subjective cure rates ranging from 43% to 92% (Ford et al., 2015).

Regenerative medicine, whose objective is the restoration or rejuvenation of tissues using endogenous or exogenous replacement stem cells, appears as a therapeutic alternative in SUI. Mesenchymal stem cells (MSC) are self-renewing adult stem cells that retain multilineage differentiation capacity. Their long-term *in vitro* expansion, homing capacity, and immunomodulatory and immunosuppressive effects have made them one of the most exciting sources for stem cell research and therapy (Torre et al., 2018; Merimi et al., 2021). MSC are able to replace cells in damaged tissue, although it is thought that they mainly exert their therapeutic action by a paracrine effect, that is, through secreted factors that will influence the response mechanisms of cells in the tissue (Jimenez-Puerta et al., 2020). There are many sources of MSC such as bone marrow, adipose tissue, or perinatal derivatives, among others (Ledesma-Martinez et al., 2016; Torre and Flores, 2020; Gao et al., 2021; Krawczenko and Klimczak, 2022). MSC transplantation has been tested in preclinical SUI models and has been shown to be safe, feasible, and effective in repairing tissue deficiencies (Sima and Chen, 2020; Maiborodin et al., 2022). MSC from different tissues have been tested in these preclinical models such as from bone marrow (Janssen et al., 2019), adipose tissue (Cui et al., 2018; Ni et al., 2018), dental pulp (Zordani et al., 2019), urine (Wu et al., 2019), and muscle (Bilhar et al., 2018; Cui et al., 2018). The safety of MSC transplantation has been sufficiently demonstrated in many phase I and II clinical trials (Rodriguez-Fuentes et al., 2021), both in autologous and in allogenic use, and there are several off-the-shelf MSC-based drug products approved by regulatory agencies in different countries (Levy et al., 2020). Periurethral injection of autologous adipose-derived MSC has been used for treatment of incontinence (Gotoh et al., 2020) and women (Kuismanen et al., 2014; Garcia-Arranz et al., 2020), proving to be a safe and effective treatment option. Among the perinatal derivatives that are source of MSC are the placenta, the umbilical cord and the amniotic membrane. The cells obtained from these perinatal tissues that are discarded after delivery, are unlimited, easy to obtain, and without ethical concerns (Silini et al., 2020). Perinatal cells obtained from the maternal layer of the term human placenta, i.e. the decidua, were phenotypically characterized as mesenchymal cells and named as decidua mesenchymal stromal cells (DMSC) (Macias et al., 2010). DMSC are a homogeneous cell population endowed with a remarkable capacity for proliferation and differentiation in culture (Macias et al., 2010; Cerrada et al., 2014; Serrano et al., 2021). These cells have been used in various animal models of disease where it has been shown that their therapeutic action is due to their migration capacity and inhibitory action on mammary tumor cells (Vegh et al., 2013), or to their immunomodulation capacity in the experimental model of autoimmune encephalomyelitis (EAE) (Bravo et al., 2016). Furthermore, DMSC are considered a safe product for allogenic uses given their hypoimmunogenic profile (Macias et al., 2010). DMSC have important advantages over adipose-derived MSC, which are the most commonly used MSC for UI. These advantages include availability, accessibility, easier to obtain, higher yield, naivety, and unique immune-modulatory properties (Macias et al., 2010; Hass et al., 2011; Silini et al., 2020; Flores et al., 2022). The aim of this study was to use DMSC to study their suitability as a treatment for SUI using *in vivo* and *in vitro* models. For *in vivo* studies, we have transplanted DMSC in the periurethral region of female rats that have been subjected to vaginal distention as a model of SUI caused by childbirth and we studied their effect on incontinence. For *in vitro* studies, we isolated suburethral connective tissue cells from patients with SUI and used them as an *in vitro* model to study the interaction between DMSC and these cells with the aim of identifying the mechanisms involved in DMSC-induced tissue repair.

## 2 Materials and methods

### 2.1 Animal model of stress UI caused by childbirth

Vaginal distention in female Sprague-Dawley rats was used to simulate the maternal injuries of childbirth. Animal experiments were carried out in compliance with the animal protection requirements and the approval of the Animal Welfare Ethics Committee at our institution. Twelve virgin female Sprague Dawley rats (Janvier Labs), 17 weeks old and weighing 300–350 g were used for this study. They were acclimatized for 14 days before starting the procedures in conditions of 12-h light-dark cycles. The rats were anesthetized using a combination of inhaled isoflurane and intraperitoneal ketamine-xylazine (37.5 and 5 mg/kg, respectively) and supplemented as needed. Vaginal distention (VD) was performed as previously described (Pan et al., 2007). Briefly, vagina was first accommodated using lubricated bouge dilators (24 F–32 F, Uterine Dilator Hegar, MEDICON). VD was performed *via* vaginal insertion of a modified lubricated 10-Fr Foley catheter and subsequent filling of the balloon with 2 mL of phosphate-buffered saline (PBS) for half an hour to let the vagina accommodate and then complete up to 3 ml. The rats were placed in anti-Trendelenburg position and the balloon was kept inside the vagina for 6 h to simulate childbirth injuries (Phull et al., 2011). Animals were monitored during the procedure using a pulse oximeter (UT100V Veterinary Pulse Oximeter; Utech Co., Ltd.). Before the end of the VD procedure and for two additional days, subcutaneous buprenorphine (.05 mg/Kg) was administered every 12 h for pain control. One week after VD, the animals were randomly distributed into two groups, control and DMSC-treated animals (*n* = 6 in each group). The treated group received two doses of DMSC resuspended in Hank´s balanced salt solution (HBSS) (2 × 10^6^ DMSC per dose and 1 week apart) while control group received two injections of the same volume of HBSS without cells. DMSC/HBSS were administered into the periurethral region by bilateral injections at the 3 and 9 o’clock positions. UI was assessed by measurement of the leak point pressure (LPP). To evaluate DMSC´s engrafment, the cells were labeled with VivoTrack™ fluorescent labeling dye (PerkinElmer, Tres Cantos, Madrid, Spain) before injection. VivoTrack 680 is a near-infrared (NIR) fluorescent lipophilic dye that intercalates in the cell membrane. Viability and percentage of stained DMSC was tested before injecting the cells using a BD FACSCalibur instrument (Becton Dickinson, San José, CA, United States). VivoTrack-labeled DMSC were injected into the periurethral area and NIR fluorescent imaging was performed by Bruker *In Vivo* Xtreme animal imaging system (Bruker, bioNova científica, Madrid, Spain) once a week during 3 weeks.

### 2.2 Measurement of leak point pressure (LPP)

Under anesthesia with inhaled isoflurane (5% for induction; 2% for maintenance), the LPP measurement was conducted as previously described (Damaser et al., 2003). Briefly, a polyethylene catheter (PE-50 Instech Laboratories, Inc.) was inserted transurethrally into the bladder that was forced to emptying. Next, to determine its capacity, the bladder was filled with PBS mixed with methylene blue until urine leakage was observed at the urethra meatus. Three filling cycles were performed to calculate the average capacity. For the Leak Point Pressure (LPP) measurement, the bladder was filled to 50% of capacity with PBS plus methylene blue. The PE-50 catheter was connected to a data acquisition device (MP36R Research System, Biopac Systems Inc.) which was used in combination with an acquisition and analysis software (AcqKnowledge Software) to record the intravesical pressure. LPP was determined by manually applying abdominal pressure to record the minimum pressure exerted to cause leakage through the urethra (Damaser et al., 2003). LPP was measured three times for animal and the average pressure was considered.

### 2.3 Histological analysis

At the end of the study, urethras were harvested and placed in a formalin fixative solution for 48 h before embedding in paraffin. 4 μm slices were used for Hematoxylin and Eosin (H&E), Masson’s trichrome and elastin van Gieson (EVG) staining. Masson’s trichrome-stained slides were evaluated by two blinded investigators for semi-quantitative analysis of tissue integrity using a 1–4 grading scale ranging from significant tissue disruption to normal tissue, as previously described (Dissaranan et al., 2014). Two blinded investigators also used Masson’s trichrome-stained slides to determine the total number of muscle fibers in the external urethral sphincter.

### 2.4 Isolation and culture of cells

SUI patients, non-SUI patients, and pregnant women were recruited at the Department of Obstetrics and Gynecology under informed consent approved by the Ethics Committee. All steps of tissue processing were performed in a biosafety cabinet using appropriate aseptic techniques.

#### 2.4.1 Isolation of cells from suburethral tissue from SUI and non-SUI patients

A total of 24 women with a median age of 56 (age range 43–69 years) with a diagnosis of mild to severe SUI were included in the study. Three gynecology patients without urinary incontinence were used as controls. Suburethral tissues were obtained by biopsy and cell cultures were established by overnight enzymatic digestion with 100 units/µL of collagenase type II (GIBCO). The cells were collected by centrifugation at 250xg for 10 min and washed twice with HBSS. The cellular pellet was resuspended in complete culture medium consisting of DMEM with 10% fetal bovine serum (Biowest, LabClinics), 1% non-essential aminoacids and 1% penicillin/streptomycin, and seeded in a 24-well plate. Cells were maintained at 37°C in 5% CO_2_ until passage 11–13.

#### 2.4.2 Isolation and culture of decidua mesenchymal stromal cells (DMSC)

Human placentas were obtained during natural or cesarean births and decidua-derived mesenchymal stromal cells (DMSC) were isolated from placental membranes by trypsin/EDTA enzymatic digestion as we described previously (Macias et al., 2010). The phenotype of DMSC was characterized by flow cytometry as we described previously (data not shown) (Macias et al., 2010; Serrano et al., 2021). Isolated cells were cultured in DMEM supplemented with 10% fetal bovine serum, 2 mM glutamine, .1 mM sodium pyruvate, 55 μM β-mercaptoethanol, 1% non-essential amino acids, 1% penicillin/streptomycin, and 10 ng/ml epidermal growth factor (EGF). For the experiments, cells from different donors have been used individually in passages between 2 and 7.

### 2.5 Quantitative real-time PCR

RNA extraction was performed using NZY Total RNA Isolation kit (NZYTech, Lda., Lisboa, Portugal) according to the manufacturer’s protocol. RNA concentration and purity were determined by spectrophotometry in Nanodrop One (Thermo Fisher Scientific, Madrid, Spain) and .5 μg of the total RNA were retro-transcribed using the High Capacity cDNA Reverse Transcription kit (Applied Biosystem, Thermo Fisher Scientific, Madrid, Spain). Real-time PCR was performed using the ABI 7500 Fast Sequence Detection System (Applied Biosystems, Thermo Fisher Scientific, Madrid, Spain) and SYBR green qPCR Master mix (Promega Biotech Ibérica SL). The PCR primers and the size of the amplified products are in Table 1. TATA-binding protein (TBP) was used as an endogenous reference for normalization of the target gene quantity by mean of the differences of Ct or threshold cycle (dCt). The 2^-dCt indicates the relative amount of the target gene respect to the housekeeping, in each sample. The 2^-ddCt indicates fold-change of the gene expression respect to a basal condition.

**TABLE 1:** Real-time quantitative PCR primers and length of products.

α-SMA	Forward sequence (5′to 3′): GTG​TTG​CCC​CTG​AAG​AGC​AT	109 bp
	Reverse sequence (5′to 3′): GCT​GGG​ACA​TTG​AAA​GTC​TCA	
Vimentin	Forward sequence (5′to 3′): GCA​GGA​GGC​AGA​AGA​ATG​GT	174 bp
	Reverse sequence (5′to 3′): CGT​TCC​AGG​GAC​TCA​TTG​GTT	
Desmin	Forward sequence (5′to 3′): GGA​CCT​GCT​CAA​CGT​GAA​GA	132 bp
	Reverse sequence (5′to 3′): GGG​CTG​GTT​TCT​CGG​AAG​TT	
Smoothelin	Forward sequence (5′to 3′): GAG​CAG​ACC​CGA​GTG​AAC​AA	111 bp
	Reverse sequence (5′to 3′): CGT​GCT​CTG​ATC​CAG​CAT​CT	
Tenascin	Forward sequence (5′to 3′): GTC​TCA​GGG​TCA​TTC​ACC​ACA	149 bp
	Reverse sequence (5′to 3′): TCT​GGC​ACT​TTC​TCG​CCT​G	
COL1A1	Forward sequence (5′to 3′): TGA​TGG​GAT​TCC​CTG​GAC​CT	116 bp
	Reverse sequence (5′to 3′): TCC​AGC​CTC​TCC​ATC​TTT​GC	
PDPN	Forward sequence (5′to 3′): TGA​GAA​AGA​ATG​GTT​TGT​CAA​CAG​TG	147 bp
	Reverse sequence (5′to 3′): GGC​GTA​ACC​CTT​CAG​CTC​TT	
Cadherin	Forward sequence (5′to 3′): CACCCTCAAGGGCCCCA	150 bp
	Reverse sequence (5′to 3′): TTA​GCT​TCT​TCT​TCA​CCC​ATT​GGA	
TBP	Forward sequence (5′to 3′): TGC​ACA​GGA​GCC​AAG​AGT​GAA	132 bp
	Reverse sequence (5′to 3′): CAC​ATC​ACA​GCT​CCC​CAC​CA	
CDKN2A	Forward sequence (5′to 3′): GAA​GGT​CCC​TCA​GAC​ATC​CCC	94 bp
	Reverse sequence (5′to 3′): CCC​TGT​AGG​ACC​TTC​GGT​GAC	
CDKN1A	Forward sequence (5′to 3′): GAC​TCT​CAG​GGT​CGA​AAA​CGG	84 bp
	Reverse sequence (5′to 3′): CTT​CCT​CTT​GGA​GAA​GAT​CAG​CC	

### 2.6 Cell contraction assay

To study the ability of suburethral isolated fibroblasts to *in vitro* reorganize and contract collagen matrices we used the collagen-based Contraction Assay Kit (Cell Biolabs, bioNova, Madrid, Spain) following the manufacturer´s instructions but with some modifications. Briefly, 1 × 10^5^ suburethral cells were suspended in 15 μl of DMEM and mixed with 80 μl of the collagen solution (1 × 10^6^ cells/mL). The collagen/cell mixture was distributed into 96-well plates and incubated for 1 h at 37°C. Immediately after collagen polymerization, 180 μL of complete culture medium was added on top of each collagen gel lattice. Cultures were incubated for 16 h, allowing stress to develop. To initiate contraction, collagen gels were carefully detached from the edges of the culture wells with the help of a needle, releasing the accumulated mechanical load. The bottom of the culture plates containing the gels were scanned at different times after the release (Canon image RUNNER ADVANCE 4245i). The diameter of each gel surface was measured with the ImageJ program (National Institutes of Health, Bethesda, MA, United States). The contraction index is expressed as percentage of the total area of the well that is occupied by the collagen gel.

### 2.7 Senescence-associated beta galactosidase assay (SA-β-gal)

SA-β-gal assay was performed as we have described previously with some modifications (Torre et al., 2021). Briefly, cells were fixed with 2% formaldehyde (vol/vol) and 0.2% glutaraldehyde (vol/vol) for 5 min, washed twice with PBS, and stained with SA-β-gal solution overnight at 37°C. Next, cells were washed twice with PBS and visualized and photographed using a Leica DMIL microscope (Leica Microsistemas S.L.U., L’Hospitalet de Llobregat, Spain).

### 2.8 Immunofluorescence staining

Cells were seeded at density of 7 × 10^3^ cells/well in 8-well Lab-Tek chamber slides (Nunc, Thermo Fisher Scientific, Madrid, Spain). After 48 h of culture, cells were fixed with 10% buffered-formalin for 15 min at room temperature, permeabilized with .5% Tween-20 in PBS for 10 min, and incubated with 5% horse serum to block non-specific binding. Primary antibodies (1:50 dilution) were incubated overnight at 4°C, washed with PBS twice and incubated with 1:200 dilution of fluorescein isothiocyanate (FITC)-conjugated or tetramethylrhodamine (TRITC)-conjugated secondary antibodies (Jackson Immunoresearch Laboratories, Vitro SA, Madrid, Spain). Nuclei were counterstained with .2 mg/ml 4′,6′diamidino-2-phenylindole (DAPI) (Sigma-Aldrich Quimica SA, Madrid, Spain) for 1 min. Cells were visualized by a Zeiss LSM 510 Meta Inverted Confocal Microscope (Carl Zeiss Meditec Iberia SAU, Madrid, Spain). The primary antibodies used were rabbit polyclonal IgG anti-αSMA (NBP1-35269, Novus Biologicals, bioNova científica, Madrid, Spain), rabbit monoclonal IgG anti-vimentin (NBP2-12472, Novus Biologicals, bioNova científica, Madrid, Spain), mouse monoclonal IgG1 anti-desmin (NB110-60508, Novus Biologicals, bioNova científica, Madrid, Spain) and mouse monoclonal IgG2b κ anti- Smoothelin (sc-376902, Santa Cruz Biotechnology, Quimigen S.L., Madrid, Spain).

### 2.9 Transwell migration assay

*In vitro* DMSC migration was determined using Millicell 24-well transwell Boyden chambers with 8 μm pore polycarbonate membranes (Merck Millipore, Spain) as we previously described (Vegh et al., 2013; Paris et al., 2016; Paris et al., 2017). Briefly, 2 × 10^5^ DMSC in 100 μl of migration media (DMEM supplemented with 55 μM b-mercaptoethanol, 1% non-essential amino acids and without FBS) were seeded in the upper chamber of the transwell system and 5 × 10^4^ suburethral tissue cells were seeded on the well below. Migration medium without cells was used as a negative control. The migration was assessed at 24 h by the CytoSelect 24-Well Cell Migration Assay (8 μm, Colorimetric, Cell Biolabs, bioNova, Madrid, Spain) following the manufacturer´s instructions. Migratory cells were stained with the Cell Stain Solution and the color was subsequently extracted with the Extraction Solution, and quantified by absorbance at 560 nm using an Enspire 2300 plate reader (PerkinElmer, Tres Cantos, Madrid, Spain). All experiments were done in triplicate.

### 2.10 Co-culture effect of DMSC on suburethral cell proliferation

To determine the *in vitro* effect of DMSC on the proliferation of suburethral cells, transwell culture inserts with .4 μm pore polycarbonate membranes and tissue cultured treatment (Merck Millipore, Spain) were used. Suburethral cells were cultured in 24 well plates at 3 × 10^4^ cells per well and DMSC were seeded onto transwell inserts at 1.5 × 10^4^ cells per insert. After 24 h, DMSC seeded onto transwell inserts were transferred to the 24-well plate of suburethral cells for co-culturing. As a control, suburethral cells without DMSC in inserts were prepared. Twenty 4 hours later, the inserts and the culture medium were removed and the viability of suburethral cells was evaluated by PrestoBlue cell viability assay (Invitrogen, Fisher Scientific, Madrid, Spain) following the manufacturer’s instructions. Briefly, 200 μl of fresh medium containing .1 mg/mL of PrestoBlue was added to the wells that were incubated for 1 h. Then, 100 μl of this medium was transferred to a black flat-bottom plate for fluorescence readings at 570 nm and 600 nm using a plate reader (Enspire 2300; PerkinElmer, Tres Cantos, Madrid, Spain). Assays were performed in triplicate, and at least twice with cells isolated from five different patients. Fluorescence of cocultured suburethral cells was expressed as a percentage relative to the fluorescence of suburethral cells cultured without DMSC which was set as a value of 100.

### 2.11 Multiplex assay to measure the co-culture effect of DMSC on suburethral cell secretion

To determine the *in vitro* effect of DMSC on the secretome of suburethral cells, Transwell culture inserts with .4 μm pore polycarbonate membranes and tissue cultured treated (Merck Millipore, Spain) were used. Suburethral cells were cultured in 24 well plates at 3 × 10^4^ cells per well and DMSC were seeded onto Transwell inserts at 1.5 × 10^4^ cells per insert. After 24 h, DMSC seeded onto Transwell inserts were transferred to the 24-well plate for co-culturing with suburethral cells in DMEM supplemented with 2% FBS. As controls of the secretion, 3 × 10^4^ suburethral cells and 1.5 × 10^4^ DMSC were grown as monocultures and fresh medium was added as described above twenty four hours later, the inserts were removed and the cell supernatant containing secreted proteins was collected and centrifuged at 10.000xg for 15 min to remove cell debris. The secretome of suburethral cells co-cultured with DMSC, suburethral cells alone and DMSC alone was evaluated by a bead-based immunoassay according to manufacturer’s protocol (ProcartaPlex^TM^ Multiplex Immunoassay, Invitrogen^TM^ Life Technologies, Labclinics, Madrid, Spain) for the following soluble factors: interleukin (IL)-6, IL-8, hepatocyte growth factor (HGF), monocyte chemoattractant protein (MCP)-1, MCP-3, vascular endothelial growth factor (VEGF)-A, stromal cell-derived factor (SDF)-1, matrix metalloproteinase (MMP)-2, MMP-3, and MMP-9.

### 2.12 Statistical analysis

Data were analyzed using GraphPad Prism software v6.0 (GraphPad Software, San Diego, CA, United States). The data were expressed as the mean ± SD. Inter-group statistical significance was performed with the unpaired Student’s t-test and mean was compared by Student’s t-test, Mann-Whitney U-test, and Wilcoxon tests as appropriate. In all experiments a *p* < .05 was considered statistically significant.

## 3 Results

### 3.1 Effects of DMSC on urodynamic parameters and urethral histology in a rat model of SUI

**FIGURE 1:**
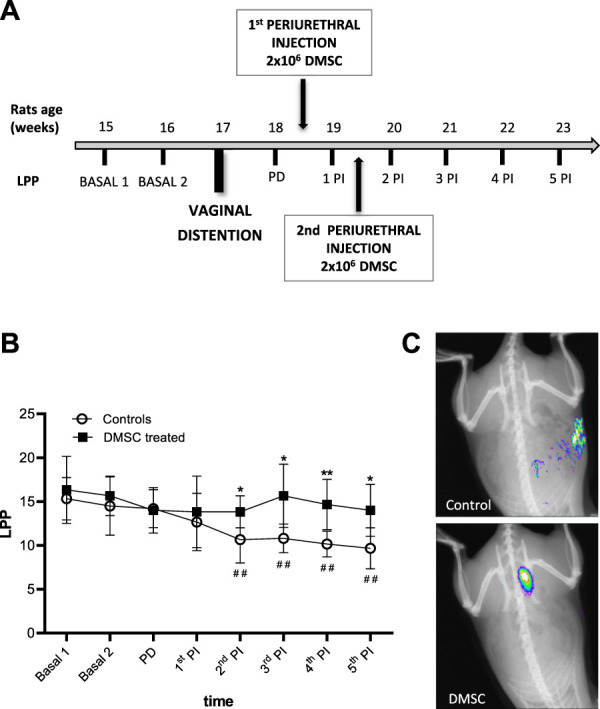
Periurethral injection of DMSC markedly prevented fall of abdominal leak point pressure (LPP) or enhanced LPP in rats after vaginal distention (VD). **(A)** Flowchart of the design of measurement of LPP, vaginal distention and treatment with DMSC. Basal 1 and Basal 2 are LPP measurements from the 2 weeks prior to VD. Post-damage (PD) is the LPP measurement after VD and before injection. Post-injection (PI) are LPP measurements after the first injection of DMSC or HBSS (controls). **(B)** Leak point pressure (LPP) measurement in DMSC-treated and control animals. DMSC prevents the drop of leak point pressures in VD rats while in control rats there is a significant drop in LPP from the third week after VD or second week post-injection. Data are shown as mean ± standard deviation (SD). *p < .05; **p < .01; ^##^ *p* < .01. Asterisks correspond to the comparison of DMSC-treated vs. control animals at each time point. The hashtag symbol indicates the comparison of control animals with respect to their baseline. **(C)** Examples of *in vivo* fluorescence images of rats transplanted with NIR-labelled DMSC or injected with HBSS (controls) 14 days after VD.

We have evaluated the regenerative role of DMSC in rats after VD. In order to maximize the damage inflicted by the distention as well as the duration of the effects, a time extended procedure has been performed as previously described (Pan et al., 2007). Baseline LPP values were determined during the 2 weeks prior to VD and the evolution of the damage was analyzed by measuring the LPP during the 6 weeks after VD (Figure 1A). To avoid bias, weekly measurements were carried out without knowing which group each individual belonged to, simulating the “double blind” technique of clinical trials. The consequence of VD was a progressive decrease in the LPP value that was evident 2 weeks after injury in control animals, i.e., those injected with HBSS (1st PI, Figure 1B, white circles), which became significant from the third week after VD or second week post-injection until the end of the study (2nd PI, Figure 1B, white circles). However, the two DMSC injections after injury prevented the decrease in LPP respect to baseline values in the treated group of animals, as shown in Figure 1B (black squares). LPP values were significantly different between DMSC-treated and control animals from the second week after the first injection (1st PI, Figure 1B) suggesting that DMSC treatment prevents loss of continence or induces recovery of continence in rats with SUI.

**FIGURE 2:**
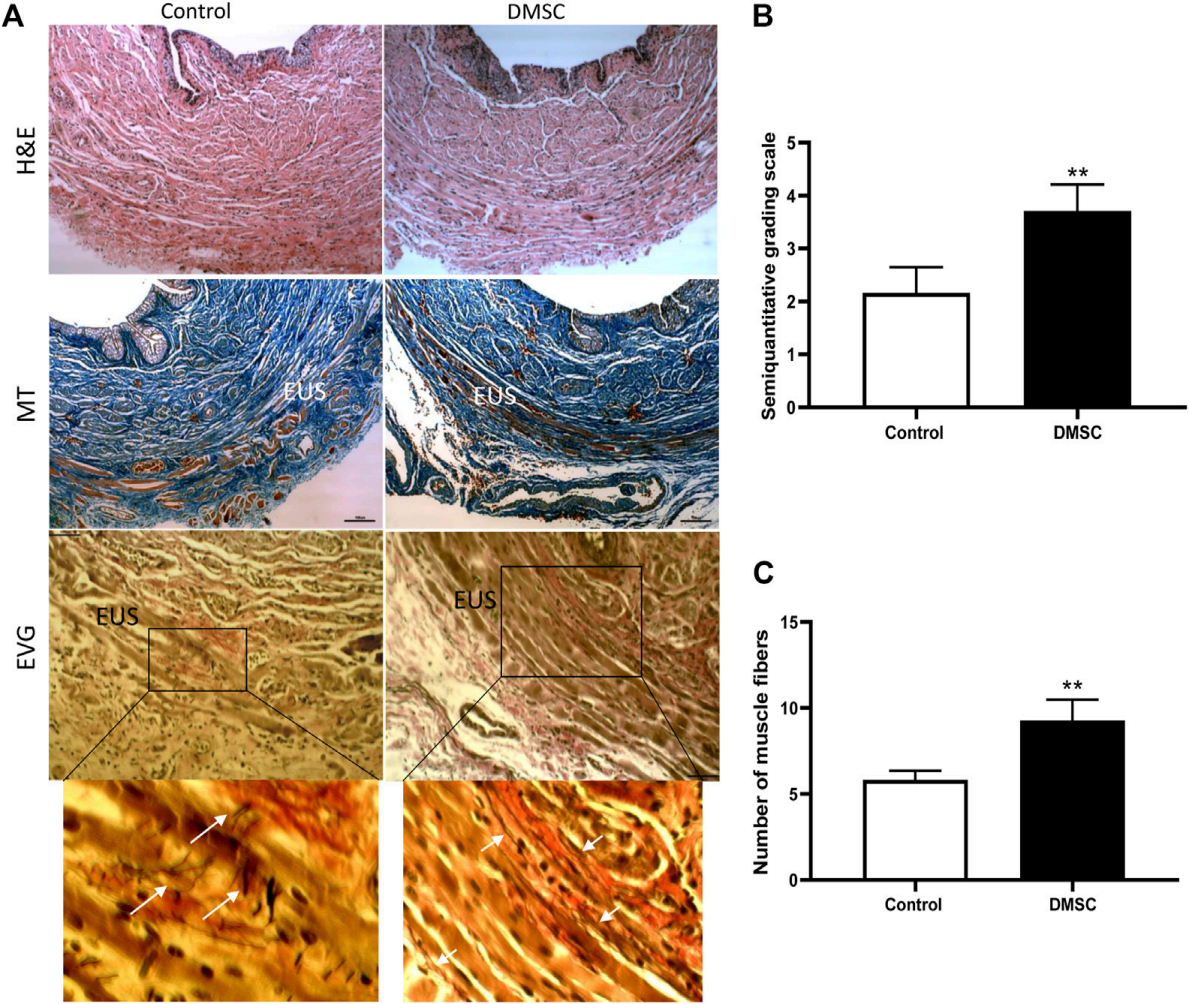
Periurethral injection of DMSC reverses VD-induced muscle and connective tissue disruption in the urethra. **(A)** Histological staining of urethral sections after VD and DMSC or HBSS (Control) treatment. Examples of urethral cross-sections stained with haematoxylin/eosin (H&E; upper panels), Masson´s trichrome (MT; middle panels) or elastin von Giesson (EVG; lower panels) 6 weeks after VD. EUS: external urethral sphincter. Inset in EVG panels is shown in higher magnification in the panels placed just below. Scale bar: 100 μm. White arrows indicate disorganized elastin fibers in Control-EVG examples while elastin fibers in DMSC-EGV examples are more organized. **(B)** Semi-quantitative analysis of Masson’s-trichrome-staining using a grading scale showing that the urethra of rats in the DMSC-treated group appeared to have more complete muscle fibers in the EUS than those in the control group. **(C)** Analysis of total number of muscle fibers in EUS in DMSC-treated and in control animals showed significant differences between the two groups. Data of B and C are shown as mean ± standard deviation (SD) and correspond to the comparison of DMSC-treated vs. control animals. **p < .01.

To assess the homing and engrafment of DMSC in the injected area, cells were stained with a near-infrared (NIR) fluorescent dye prior to injection, which allowed their visualization by means of an *in vivo* imaging system. The images showed that the DMSC remain in the ventral area for at least 14 days after the second injection, this is around 10 days before the end of the study (Figure 1C). At the end of the study, immunofluorescence analysis of urethral cross-sections using an anti-human mitochondria antibody failed to detect the presence of DMSC (data not shown). By H&E-staining of urethral sections, we did not identify any sign of inflammation due to DMSC injection suggesting that the cells are well tolerated and no adverse reactions occur, although the animals used in this study are immunocompetent (Figure 2A). To explore the mechanisms that might contribute to DMSC-mediated recovery of continence, we carried out further histological analysis of urethras using Masson’s trichrome and EVG staining. As is evident in Figure 2A, the VD involved extensive rupture of the muscle fibers of the external urethral sphincter (EUS) with marked infiltrates of ECM, as well as atrophy of elastic fibers near the EUS. Interestingly, it is shown that DMSC treatment resulted in a significant increase in the integrity (Figures 2A,B) and the number of muscle fibers in the EUS (Figures 2A,C). Furthermore, EUS showed less infiltration of ECM and the presence of larger elastic fibers in DMSC-treated animals (Figure 2A).

### 3.2 Characterization of fibroblast cells isolated from SUI patients

**FIGURE 3:**
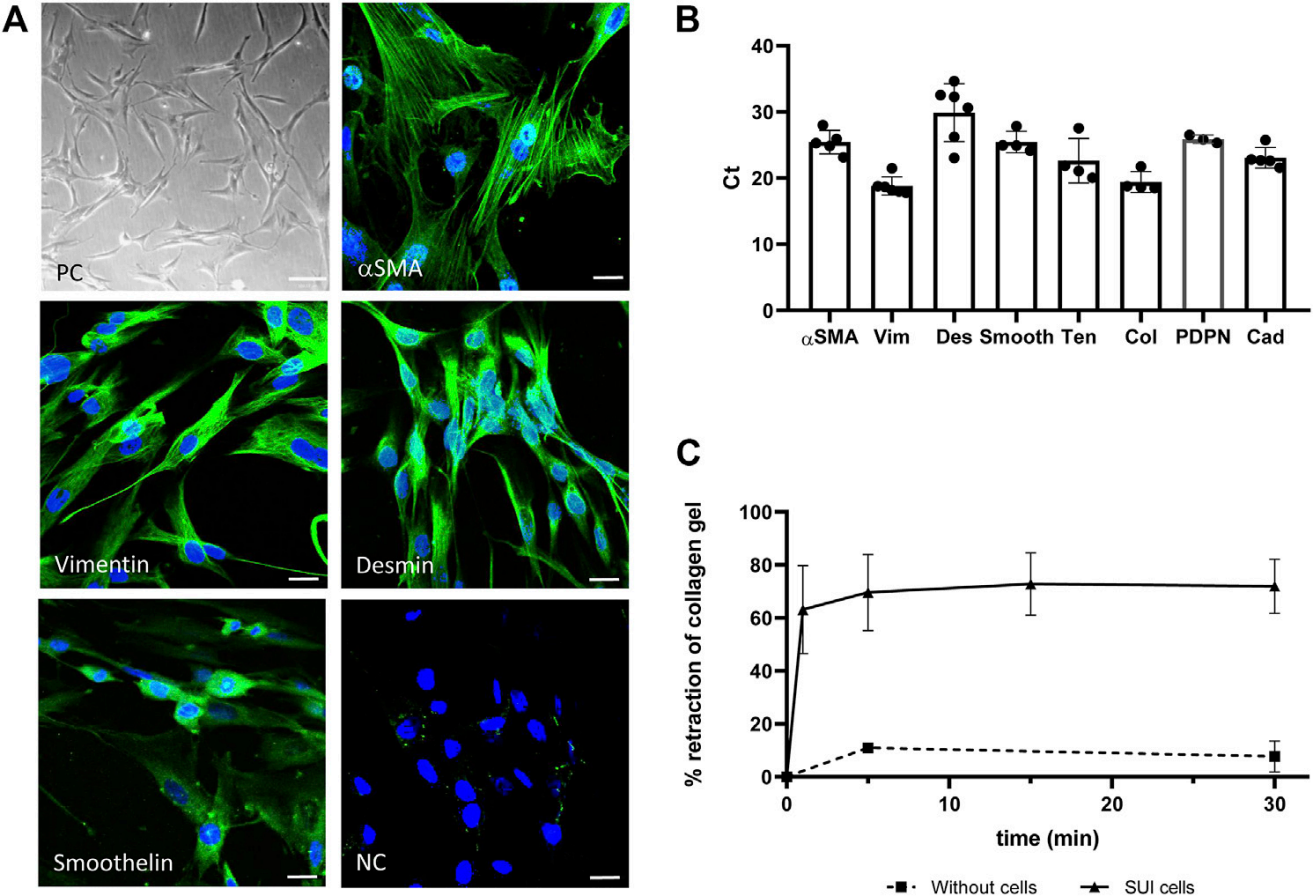
Characterization of primary suburethral myofibroblasts cultures. **(A)** Representative photomicrograph of suburethral myofibroblasts isolated from SUI patients. Cells are fibroblast-shaped as shown in phase contrast (PC) panel and express several markers of myofibroblast, such us α-SMA, vimentin, desmin and smoothelin (in green) as shown in confocal microscopy images. NC is the negative control of immunofluorescence. Bar = 100 μm in PC panel and 22 μm in immunofluorescence panels. **(B)** Gene expression of myofibroblast markers was determined by real-time qPCR. The analyzed cells correspond to passage 3. Data are presented as mean ± standard deviation (SD). Gene abbreviations: α-SMA, alpha smooth muscle actin; Vim, vimentin; Des, desmin; Smooth, smoothelin; Ten, tenascin; Col, collagen I, PDPN, podoplanin; Cad, Cadherin. **(C)** Retraction of a collagen lattice by suburethral myofibroblasts. Evolution of the diameter of collagen gel surface incubated with suburethral myofibroblasts (triangles) or without cells (squares) once the gel is detached from the edges of the culture wells. Data is calculated as the area of the well that is not occupied by the collagen gel after detachment respect to the total area of the well, and expressed as percentage.

To deepen into the cellular and molecular mechanisms by which DMSC exert their effect on the recovery of continence, an *in vitro* model was carried out using cells obtained from SUI patients. The cells were isolated from the suburethral tissue and expanded in culture. They grow on plastic as adherent fibroblast-like cells (Figure 3A) with an estimated doubling time of 72 h (data not shown). Gene and protein expression analysis, showed that SUI cells expressed alpha smooth muscle actin (α-SMA), vimentin, desmin and smoothelin, suggesting that they are myofibroblasts (Figures 3A,B). Interestingly, immunofluorescence staining showed that cells positive for α-SMA were also positive for smoothelin (results not shown). The expression of other myofibroblast markers such as podoplanin, tenascin, cadherins and collagen I, further confirmed the presence of myofibroblasts in suburethral cells isolated from SUI patients. To analyze whether myofibroblast differentiation could be associated to pelvic floor pathology, we carried out the characterization of cells from the suburethral tissue of patients without SUI (non-SUI) or other pelvic floor disorder. We found that these non-SUI cells expressed most of the myofibroblast markers previously detected in SUI cells, except for desmin (Supplementary Figure S1). Desmin expression was extremely low or almost undetectable (appearing above cycle 36 in qPCR) in non-SUI cells. When the expression of the other myofibroblast genes was corrected to the housekeeping gene (2^dCt), we found that it was higher in SUI cells, except for collagen that was higher in non-SUI cells, although the differences were not statistically significant (data not shown).

Contractility is a property of myofibroblasts and we determined by a collagen-based contraction assay whether SUI myofibroblasts, coming from a damaged tissue, maintained their functionality. We calculated the area of the well that is occupied by the collagen gel A) respect to the total area (A_0_) at different time points and expressed the contraction index as [1-(A/A_0_)]*100 (Figure 3C). Using this approach, we observed that SUI cells contracted the matrices by 60% within the first minute of release and reaching a maximum of 80% retraction within 5 minutes of release (Figure 3C). While contraction was significantly increased in the presence of SUI fibroblasts, control matrices without cells had no contraction. These results confirmed that SUI cells had the contractile phenotype of myofibroblast.

**FIGURE 4:**
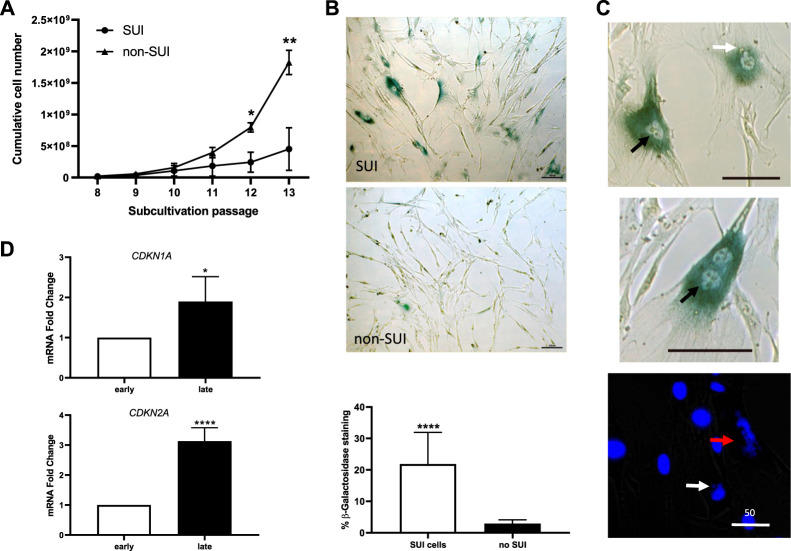
Suburethral myofibroblasts presented a limited cell proliferation and premature senescence. **(A)** Cells from SUI myofibroblast cultures (*n* = 5) or non-SUI (*n* = 3) fibroblast cultures were counted at each passage and the cumulative cell number was determined. Error bars are SD of mean; (*p < .05, **p < .01). **(B)** Representative photomicrograph of blue-colored staining for SA-β-gal activity in cells from SUI myofibroblast cultures or non-SUI cultures. The images correspond to cell passage 9. The graphic represents the percentage of SA-β-gal positive cells (blue) cells calculated respect to the number of total cells found in 10 and 15 fields of SUI (*n* = 2) and non-SUI (n = 3) cell cultures, respectively. **(C)** Analysis of senescence markers in SUI myofibroblast in early and late passages, 3 and 11, respectively. Change in CDKN1A and CDKN2A mRNA expression (*n* = 5). Error bars are SD of mean; (*p < .05, ****p < .0001). **(D)** High magnification of senescent cells found in SUI cultures, showing irregular nuclei (black arrow, upper panel), blebbing nuclei (white arrow, upper and lower panel), broken cell nucleus (red arrow), or binucleate cells (middle panel). Bar = 100 micras, except where specified to be 50 micras.

Growth of SUI myofibroblast cultures was analyzed (Figure 4A). Cells at different passages were counted and the number of accumulated cells was calculated. An exponential growth was observed until passage 10 when SUI cells almost stopped dividing. Interestingly, cells isolated from suburethral tissue of non-SUI patients continued their exponential growth beyond passage 10, and the number of cells accumulated was significantly higher from passage 12 onwards (Figure 4A). Morphological changes associated with a senescent phenotype, such as flattened and enlarged cells, were visible in high-passage SUI myofibroblast cultures compared to cells from no incontinent women (non-SUI cells) (Figure 4B). SUI cells exhibited significantly more cells positive for the marker of senescence SA-β-gal. (Figure 4B). It is remarkable the presence of binucleated cells and other abnormalities associated with aging affecting the morphology of the nuclei in SUI SA-β-gal positive cells such as enlargement, irregular shape, invaginations, blebbing in nuclear margin, and even total loss of structure (Figure 4C). Furthermore, another feature of senescence in late passage SUI cells was the significant upregulation of the genes encoding p16 and p21 cell cycle regulators, CDKN2A and CDKN1A, compared to early passage (Figure 4D).

### 3.3 Effect of co-culture on the proliferation, migration and paracrine response of DMSC and suburethral myofibroblast cells from SUI patients

**FIGURE 5:**
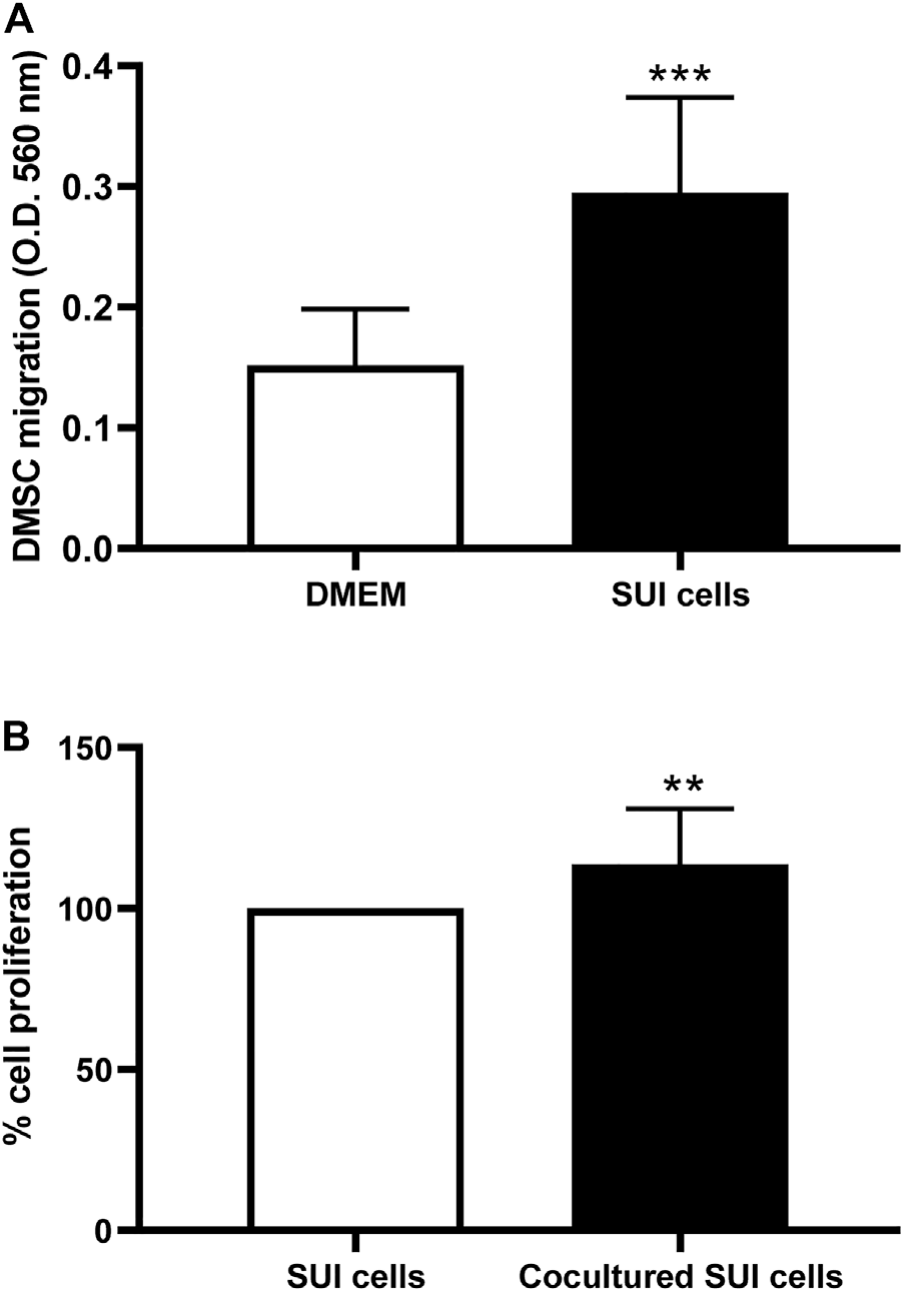
Co-culture of DMSC and SUI myofibroblasts has effects on the behavior of both cell types. **(A)** *In vitro* transwell migration assay of DMSC toward suburethral myofibroblasts or culture medium. Suburethral myofibroblasts enhanced the migration ability of DMSC *in vitro*. Data is expressed as mean ± SD, *n* = 5 in duplicate, ***p < .001. **(B)** Proliferation of suburethral myofibroblasts was determined by Presto blue assay after co-culture with DMSC seeded on Transwell® culture inserts. DMSC enhanced the proliferation of suburethral myofibroblast *in vitro*. The cells analyzed correspond to passages 5–6. Data is expressed as Mean ± SD, *n* = 5, **p < .01.

To identify the mechanisms that could be involved in the regenerative action of DMSC in SUI pathology, we performed several *in vitro* assays to study the interaction between SUI myofibroblast and DMSC. First, we tried to find out whether SUI myofibroblasts exert *in vitro* chemoattraction on DMSC, given that, as we previously reported using *in vitro* and *in vivo* studies, DMSC migrate and home at sites of injury (Vegh et al., 2013; Paris et al., 2016). As it is shown in Figure 5A, our results indicate that DMSC migrated significantly towards SUI myofibroblasts compared to their migration towards complete culture media used as a negative control.

To study the soluble factors secreted by SUI myofibroblasts that might be involved in the DMSC migration, we used a multiplex immunoassay. The results showed that the secretome of SUI myofibroblasts contained several chemokines known to regulate the recruitment of immune cells during the inflammatory phase of wound healing, such as SDF-1, MCP-1, and MCP-3 (Table 2). These results would suggest that suburethral myofibroblast obtained from patients with SUI probably present some type of damage that would give rise to a pro-inflammatory environment capable of promoting the migration and homing of DMSC.

**TABLE 2:** Chemokine analysis in secretome of SUI myofibroblasts by multiplex assay (*n* = 6).

Chemokines	Concentration [pg/mL]
SDF-1	1759.31 ± 395.25
MCP-1	1482.16 ± 189.81
MCP-3	22.84 ± 6.56

**FIGURE 6:**
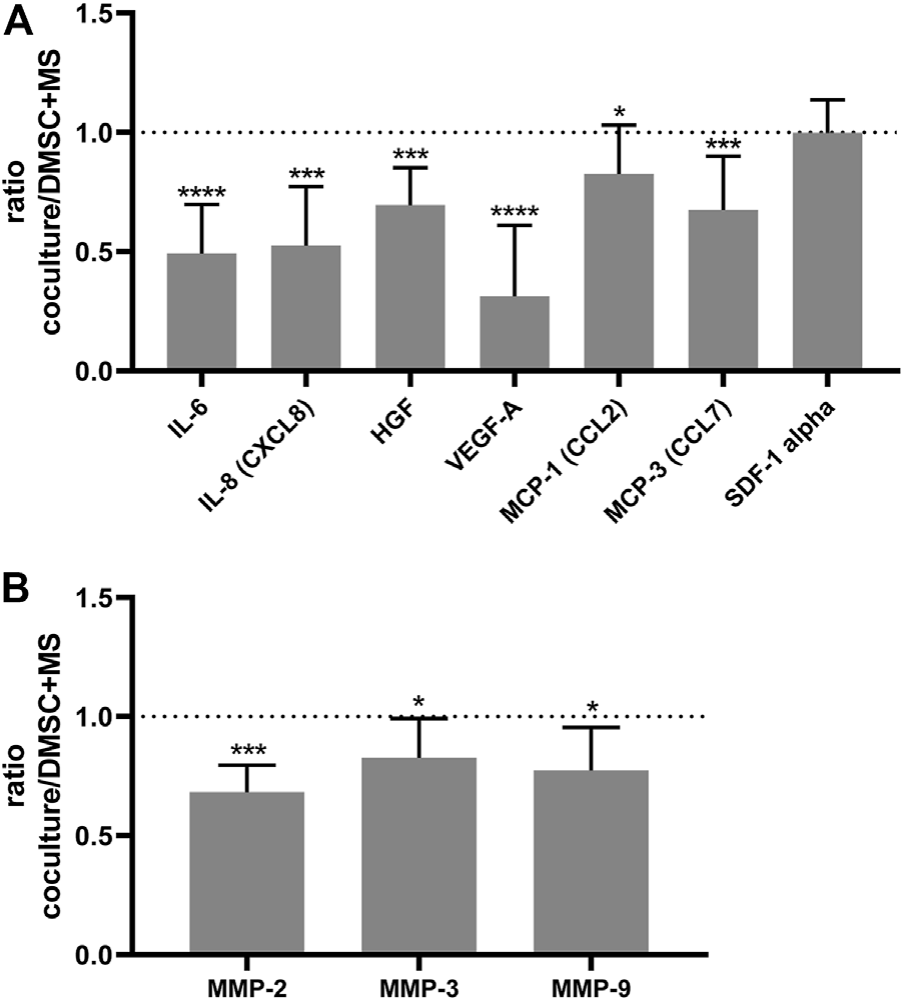
DMSC exert a paracrine action that modulates the secretion of soluble factors by SUI myofibroblasts. **(A)** Ratio of the amount measured by Luminex of IL-6, IL-8, HGF, MCP-1, MCP-3, and VEGF in the coculture of DMSC and suburethral myofibroblast cells relative to the amounts in the monocultures (*n* = 6). **(B)** Ratio of the amount measured by Luminex of MMP-2, MMP-3, and MMP-9 in the co-culture of DMSC and suburethral myofibroblast cells relative to the amounts in the monocultures (*n* = 5). The cells analyzed correspond to passages 5–6. These factors are involved in inflammation, angiogenesis, ECM-remodeling, and chemoattraction.

Next, to assess whether DMSC exert their effect through a paracrine action, that is, through the secretion of soluble factors that could alter the behavior of SUI myofibroblasts, transwell co-cultures between SUI myofibroblast and DMSC were established. We first analyzed whether DMSC have an effect on the proliferation of SUI myofibroblasts (Figure 5B). It was evident that co-culture with DMSC significantly induced SUI myofibroblast proliferation compared to the cells cultured alone (Figure 5B). Cytokines and growth factors in the secretome of the SUI and DMSC cocultures, as well as of both separate cell cultures, were analyzed by multiplex protein assay (Figure 6). This assay showed that co-culture of SUI myofibroblasts and DMSC resulted in a significant decrease in the levels of the pro-inflammatory cytokines IL-6 and IL-8 when expressed as a ratio respect to their secretion by both cell types cultured separately (Figure 6A). Likewise, a significant decrease was observed in the levels of two angiogenesis-related growth factors, such as HGF and VEGF-A, and of the chemoattractant proteins MCP-1 and MCP-3 (Figure 6A). MMP-2, MMP-3, and MMP-9 were also found to be significantly decreased in DMSC and SUI myofibroblast co-culture when analyzed relative to the levels found in both cell types when cultured separately (Figure 6B). MMPs are enzymes that degrade extracellular matrix proteins and are also involved in various cell functions, such as cell proliferation, migration, differentiation, apoptosis, angiogenesis, and host defense. All factors analyzed have been associated with the cellular senescence phenotype and linked to the development of diseases. Our results suggested that DMSC would ameliorated SUI through a paracrine action acting on the proliferation and the secretion of senescence-related factors by SUI myofibroblasts.

## 4 Discussion

Several mechanisms have been described by which childbirth predisposes women to develop pelvic floor pathologies, including mechanical damage that affects the pelvic floor support systems, denervation, ischemia and reperfusion injury, and defective soft tissues remodeling, among others (Memon and Handa, 2013). VD in rats is a validated animal model of birth-related trauma that approximates the consequences of vaginal delivery on pelvic support tissues and loss of continence in women (Cannon et al., 2002). In these animal model, it is described that the inflation of a balloon to distend the vagina causes damage to nearby organs and tissues over a prolonged period of time (Hijaz et al., 2008). VD produces a decrease in blood flow and hypoxia in the urogenital organs, that is bladder, urethra and vagina (Damaser et al., 2005), as well as important histological abnormalities such as a reduction in the content of collagen, disorganization and fragmentation of elastic fibers, and a shortening of striated muscle fibers (Wang et al., 2011; Dissaranan et al., 2014). Functionally, VD produces a decrease in urethral resistance and development of SUI (Cannon et al., 2002). Although VD is a good model to recapitulate the birth injury, this animal model has the limitation that the functional, structural and biomechanical defects have a short durability, and some kind of recovery occurs between 10 days and 6 weeks, depending on the time of VD (Herrera-Imbroda et al., 2015). For that reason, we have used a 6-h VD procedure to provide maximum damage (Pan et al., 2007) and placed the animals in a reverse Trendelenburg position so that the pressure of the inflated balloon within the vagina simulates delivery (Phull et al., 2011). Our results show that 6 weeks after VD, LPP has not yet recovered in control animals. It would be very interesting to carry out additional studies in the future to see the long-term effects of both the damage and the therapeutic effect of DMSC.

In this study we have evaluated the regenerative role of perinatal mesenchymal stromal cells obtained from the decidua (DMSC) in the described rat VD model. Our results showed that periurethral transplantation of DMSC 1 week after prolonged VD preserved urethral functionality by preventing LPP drop, which was however significant in untreated animals from the second week after injury. Labeled DMSC were detected in the lesion for up to 14 days, but there was no signal thereafter, probably due to limited survival of xenotransplanted cells in immunocompetent animals (Dai et al., 2015). Histological analysis of the urethras at the end of the study, i.e. six weeks after VD, showed that DMSC treatment increased the integrity of the muscle fibers of the EUS, in which little ECM infiltration and larger elastic fibers were found, compared with the extensive disruption of the muscle fibers found in the EUS of untreated animals. MSC isolated from muscle (Cui et al., 2018; Bortolini et al., 2019), urine (Wu et al., 2019), and adipose tissue (Cui et al., 2018; Ni et al., 2018) have also been used experimentally in the VD animal model of SUI, showing that MSC induced morphological and histological regeneration and improved urinary function in rats through a paracrine process (Sima and Chen, 2020). This paracrine effect could be stimulated, at least in part, by the hypoxic microenvironment generated by the VD. It has been demonstrated that hypoxia stimulates the paracrine action of MSC, their proliferation and migration (Yang et al., 2022). To our knowledge, this is the first demonstration of the use of perinatal MSC in the treatment of experimental SUI.

To understand the mechanisms of action underlying the therapeutic effects and *in vivo* behavior of DMSC, we have used an *in vitro* model based on cells isolated from suburethral tissue of SUI patients. First, we have characterized these SUI-isolated cells to better understand the physiopathology of SUI and, second, we have studied their interaction with DMSC when cocultured. Cells isolated from SUI patients are adherent fibroblast-like cells with an estimated doubling time of 72 h. According to expression analysis we conclude that suburethral SUI cells are myofibroblasts expressing several characteristic markers such as the α-SMA, and the cytoskeleton proteins, vimentin, and desmin. While α-SMA expression is considered the most reliable marker of myofibroblasts (Serini and Gabbiani, 1999) and vimentin is an intermediate filament of stromal cells, desmin expression has been associated with the presence of lesional myofibroblasts (Skalli et al., 1989). The colocalization of vimentin and desmin has been previously described in the immunohistochemical analysis of vaginal wall tissue from patients with SUI (Wen et al., 2012). Here we have also found that SUI myofibroblast express other myofibroblast markers such as smoothelin, podoplanin, tenascin, cadherin and collagen I. This is the first study to show the expression of these myofibroblast markers in SUI cells although their role in the physiopathology of SUI needs to be addressed. All these genes are expressed in myofibroblasts of several chronic diseases associated with fibrosis and inflammation (Aneiros-Fernandez et al., 2011; Darby et al., 2014; Kasprzycka et al., 2015; Quintanilla et al., 2019; Chen et al., 2021). Here we have shown co-localization of α-SMA and smoothelin in SUI myofibroblast. Myofibroblasts that co-express α-SMA and smoothelin (Lepreux et al., 2013), podoplanin (Braun et al., 2011; Nazari et al., 2016; Enomoto et al., 2018; Hirayama et al., 2018), cadherin (Hinz et al., 2004; Bowler et al., 2018), tenascin (Katoh et al., 2020) or collagen I (Zhang et al., 1994) have been found during the fibroblast-to-myofibroblast transition and are associated with advanced stages of fibrosis in several diseases. All these myofibroblast markers have also been found in the control group of women, as described by others for women operated on for other gynecological indications (Ruiz-Zapata et al., 2020). A limitation of the present work is the low number of not incontinence patients, so these results need to be confirmed in the future with a higher number of samples. Furthermore, it is noteworthy that the women in the control group were not incontinent but were not very different to the SUI group in terms of parity, and vaginal deliveries. The endopelvic fascia contains fibroblasts, myofibroblasts, and other cell types, and the structural features of this tissue along with the molecular composition of the ECM are responsive to local mechanical forces (Siccardi and Valle, 2022). It has been described the presence of myofibroblasts with contraction capacity in different fascial tissues under normal conditions. These myofibroblasts regulate fascial tension and probably influence the dynamics of the supported muscle tissues (Schleip et al., 2019). Furthermore, myofibroblasts derive from the tissue fibroblasts in response to injury. The fibroblast-to-myofibroblast transition is a well-known hallmark of the pathological state of tissues and is closely related to ECM remodeling processes (D'Urso and Kurniawan, 2020). Changes in the molecular composition and/or in the mechanical properties of the ECM induce alterations in various cellular functions such as migration, proliferation, differentiation and apoptosis (Jaalouk and Lammerding, 2009). We suggest that the myofibroblast differentiation in the suburethral tissue of patients with incontinence may be part of response mechanisms to tissue damage and ECM remodeling processes after childbirth, menopause and/or tissue aging. In other pathology of the pelvic floor, pelvic organ prolapse (POP), which is etiologically related to incontinence, it has been seen that the stiffness and composition of the ECM regulate the differentiation of vaginal myofibroblasts (Ruiz-Zapata et al., 2020). Monitoring the growth of SUI myofibroblasts in culture we observed that they almost stopped dividing around the passage 10, whereas suburethral cells isolated from non-incontinent women continued to grow exponentially until at least passage 13. SUI myofibroblasts around passage 10 showed positivity for senescence-associated beta-galactosidase staining as well as increased expression of genes encoding p16 and p21, confirming the aging of SUI cells (Del Rey et al., 2019). Additionaly, the aging of the SUI culture was evident by nuclear morphological anomalies previously described as senescence hallmarks (Alicka et al., 2020; Pathak et al., 2021). Premature cellular senescence has been described as a cause of POP (Huang et al., 2020), and has also been implicated in other health issues such as miscarriage (Torre et al., 2021) or rheumatoid arthritis (Del Rey et al., 2019). Based on our observations regarding SUI cells behavior in culture we agree with other authors that cellular senescence may be one of the main pathological mechanisms underlying SUI (Alperin et al., 2022). Cellular senescence is a process that can be caused by mechanical stress (Liu et al., 2022), oxidative stress (Del Rey et al., 2019) or estrogen depletion (Wei et al., 2021), among others, and contributes to tissue aging since senescent cells negatively affect nearby cells and surrounding tissues through paracrine mechanisms. To our knowledge, this is the first demonstration of cellular senescence mechanisms associated with SUI.

SUI myofibroblasts were co-cultured with DMSC using the transwell assay to understand the molecular mechanisms of this interaction. Our results suggest that there is a paracrine interaction between DMSC and SUI myofibroblasts through soluble mediators, causing changes in their cellular behavior and secretome. DMSC induced SUI myofibroblast proliferation, as observed by the significant increase in metabolic activity of these cells cocultured with DMSC compared to SUI myofibroblasts cultured alone. This effect may be one of the therapeutic mechanisms by which DMSC enhance tissue repair of pelvic floor support tissues *in vivo*. In other published study, the culture medium of MSC isolated from bone marrow induced the proliferation of fibroblasts isolated from the anterior vaginal wall of patients with SUI (Jiang et al., 2021). Similarly, the curative effect of low-intensity extracorporeal shock wave therapy in patients with SUI (Lin et al., 2021) is attributed, among others, to a cell proliferation effect as observed in the SUI animal model induced by VD (Wu et al., 2018). Furthermore, in this cell-to-cell communication, DMSC were found to be strongly attracted to SUI cells when co-cultured. Indeed, we determine that the secretome of SUI cells contains several chemokines, such as SDF-1, MCP-1, and MCP-3 which act as chemoattractants towards immune cells during tissue repair process. Similar to leucocytes, MSC express many receptors and cell adhesion molecules that respond to chemokines released from tissues and are involved in their homing. SDF-1 and MCP-1 are some of the main chemokines that exert chemoattraction on MSC in general (Ghaffari-Nazari, 2018), and have also been identified as chemoattractants on decidua MSC (Abumaree et al., 2016). Furthermore, MCP-3 has been recognized *in vitro* and *in vivo* as a homing factor of bone marrow MSC to injured myocardium (Schenk et al., 2007). We have already shown that the therapeutic action of DMSC strongly relies on their ability to migrate into injured tissues (Vegh et al., 2013; Paris et al., 2016), and these chemokines may be responsible for the migration effect induced by SUI myofibroblast. Secretome analysis of SUI and DMSC myofibroblast co-culture by multiplex showed a significant reduction in the secretion of MCP-1 and MCP-3, and no change in SDF-1 respect to the amount secreted by these cells when grown in monoculture, suggesting a paracrine interaction between both cell types. Other authors have published *in vivo* data reporting the relevance of these chemokines in incontinence. MCP-3 has been found significantly over-expressed in urethral and vaginal tissues immediately following VD (Woo et al., 2007), and MCP-1 has been determined to be a predictive biomarker of urinary incontinence in males undergoing prostatectomy (Liss et al., 2016). In contrast, SDF-1 was significantly underexpressed in urethral and vaginal tissues after VD (Woo et al., 2007), but it has been successfully evaluated as an experimental therapy for SUI in rats after VD based on its role in neovascularization and chemotaxis of stem cells (Khalifa et al., 2020).

The coculture of DMSC and SUI myofibroblasts also showed downregulation in the secretion of IL-6, IL-8, HGF, and VEGF. Upregulation of IL-8, VEGF, MCP-1, MCP-2, or MCP-3 has been found in the female genitourinary tract immediately after VD (Wood et al., 2008). These cytokines and chemokines are secreted in inflammatory processes, but they are also part of what is known as SASP or senescence-associated secretory phenotype (Coppe et al., 2010; Lopes-Paciencia et al., 2019). SUI, as other pelvic floor disorders, is not considered an inflammatory disease but rather a multifactorial chronic disorder related to tissue deterioration. The secretion of SASP components, due to their proinflammatory effect, can be detrimental to the tissue giving rise to a degenerative phenotype and favoring the progression of the disease. For this reason, other authors have stated that both, UI and POP, are the result of degenerative changes in the tissues of the pelvic floor (Radziszewski et al., 2005; Sima and Chen, 2020), which strongly limits their ability to regenerate.

Likewise, we observed a significant downregulation of secreted MMP-2, MMP-3, and MMP-9 in our coculture study. Closely associated with cellular senescence is ECM remodeling, a process with consequences on tissue homeostasis. These phenomena feed off each other since some of the molecules that make up the SASP are MMPs or enzymes that degrade the ECM (Coppe et al., 2010; Lopes-Paciencia et al., 2019), and abnormal ECM remodeling is thought to lead to cellular senescence (Blokland et al., 2020). There is also *in vivo* data on the implication of ECM-degrading enzymes in incontinence, as increased MMP-2 and MMP-9 activity was reported in the vaginal wall after VD (Rahn et al., 2008). These MMPs degrade collagen, collagen fragments and elastin and if their expression is altered, an abnormal degradation of the ECM occurs (Cabral-Pacheco et al., 2020). A decrease in collagen and elastin content has been described in the pelvic tissue of SUI patients, suggesting that alterations in connective tissue metabolism are involved in the onset of SUI (Pantatosakis et al., 2016).

## 5 Conclusion

Periurethral injection of two doses of DMSC prevented loss of continence in rats after VD, which is an animal model that reproduces SUI caused by childbirth. Despite of the absence of prolonged DMSC engraftment, they were able to reverse the VD-induced disruption of muscle and connective tissues in the urethra, probably by paracrine mechanisms. Characterization of cells isolated from suburethral tissue of patients with SUI has reported the presence of myofibroblasts, a cell related to tissue injury that reached premature senescence in the culture. In coculture, SUI myofibroblasts significantly increase DMSC migration, and DMSC induced SUI myofibroblasts to proliferate. Currently, the molecular mechanisms underlying SUI remain unknown, but it is very likely that alterations in ECM are at the origin, since parity and aging, the main risk factors for SUI, are characterized by intense ECM remodeling. Changes in the molecular composition and/or mechanical properties of the ECM affect cellular functions and can lead to cellular senescence. Senescent cells are characterized by a permanent cell growth arrest and a senescence-associated secretory phenotype or SASP, which includes the secretion of proinflammatory and ECM-degrading factors that can be harmful to tissues. Coculture of DMSC and SUI myofibroblasts resulted in downregulation of several SASP factors. Based on these results, we hypothesized that DMSC could be a therapeutic option for SUI by counteracting the effects of senescence in damaged pelvic tissue through migration to the injured tissue, stimulation of cell proliferation, and modulation of the secretion of SASP components, which would allow the recovery of tissue homeostasis.

## Data Availability

The raw data supporting the conclusion of this article will be made available by the authors, without undue reservation.

## Supplementary material

Supplementary Figure S1 Gene expression of myofibroblast markers in suburethral fibroblasts cultures of gynecological patients without incontinence (non-SUI). Gene expression of myofibroblast markers was determined by real-time qPCR in suburethral fibroblasts isolated from patients without incontinence. The analyzed cells correspond to passage 3 (n=2 or 4). Data are presented as mean ± standard deviation (SD). Gene abbreviations are as follow: α-SMA, alpha smooth muscle actin; Vim, vimentin; Des, desmin; Smooth, smoothelin; Ten, tenascin; Col, collagen I, PDPN, podoplanin; Cad, Cadherin. Desmin does not appear in this graph because non-SUI cells do not express it
