# Supplementary figures and images for: Perinatal mesenchymal stromal cells of the human decidua restore continence in rats with stress urinary incontinence induced by simulated birth trauma and regulate senescence of fibroblasts from women with stress urinary incontinence
Source: Front Cell Dev Biol. 2023 Jan 18;10:1033080. doi: 10.3389/fcell.2022.1033080 (PMC9893794; doi:10.3389/fcell.2022.1033080)

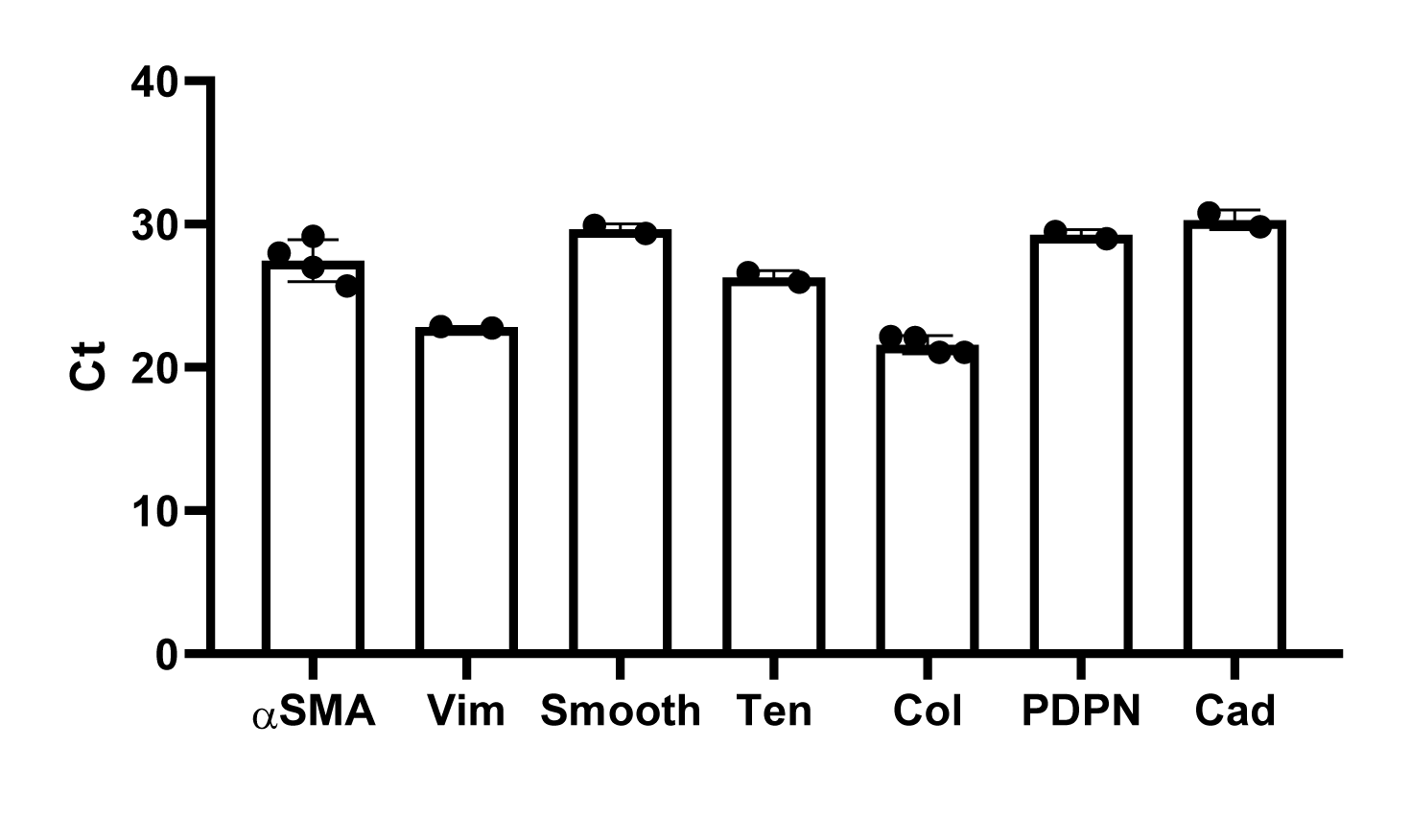

Supplement: Supplementary file 1 [file Image1.TIF]
